# The British Medical Association and Antiseptic Surgery

**Published:** 1895-07-13

**Authors:** 


### The British Medical Association and Antiseptic Surgery.

An eminent bacteriologist, well known as an experimental worker in the field of disease-producing bacilli, has recently affirmed that hardly one surgeon in London who has passed the age of fifty-five has any complete comprehension of the antiseptic system in its detailed application to the art of modern surgery. If the statement be true, it is at least surprising. From a practical point of view it is exceedingly important to find out whether it be true or not.

The British Medical Association, which is the annual parliament of British medicine and surgery, will hold its meetings in the metropolis next month. It is nearly a quarter of a century since the last meeting of the Association was held in London. The occasion is therefore rare; it is intended, if possible, to make it unique. A large sum of money has been subscribed, and will be expended freely in lending *éclat* to the proceedings. Distinguished physicians and surgeons from all parts of the world will be present by special invitation. Yet, whether by accident or design we know not, in the whole programme of the proceedings of the Surgical Section, as recently announced, there is not so much as even a casual mention of antiseptic surgery, either as a whole or in any of its details and departments. What is even more striking is the fact that the great pioneer, demonstrator and perfecter of antiseptic surgery, both as to its science and its art, is not so much as mentioned by name in the programme of the Surgical Section.

If we had been asked a week ago to give a reason for these very remarkable omissions, our reply would have been that antisepticism is so widely accepted, so thoroughly understood, and so intelligently practised by every man of any pretence to science, whether in medicine or surgery, that it is fast receding into that vast region of accepted truth which goes by the name of "commonplace." But if the judgment of our eminent bacteriologist may be relied upon, such an answer would have been the very opposite of the truth. Antiseptic surgery is not passed over by the annual parliament of the British Medical Association because it is universally received and practised, but because in practice it is not universal and in theory it is not understood. To us this explanation appears to be little short of incredible. To "an eminent bacteriologist" it is the plain and patent truth.

But, surely, if any advance in medical science was ever capable of demonstration without flaw or doubt, that advance has been demonstrated in the case of antiseptic surgery! If there be a weak link in the chain, where is it? The chemistry of antisepticism is complete, as every tyro in the study of fermentation and putrefaction is well aware; the pathology is as complete as the chemistry; and the application of the principle to wounds, accidental or artificial, great or small, new or old, external or internal, has been as successful in the hands of friends and foes, believers and agnostics, as it is possible for any human work to be. What more then is demanded? If it be as we say, that antisepticism is the greatest advance in the art of life-saving in surgery or medicine which the latter half of the nineteenth century has seen, where is the surgeon who, considering the question from the point of view alike of science and of humanity, dares to be anything but an intelligent and thorough antisepticist in every detail of his art?

The question here raised ought to be definitely brought before the annual meeting of the British Medical Association. Let us at least know where we all stand in relation to the great scientific and practical discoveries of recent times. Here we have a great truth, of first-rate importance to every man, woman, and child in the world. The natural depositaries and utilisers of this truth are all the medical practitioners in the world; but above all those who practise the separate art, and those who teach the science of surgery. Is this truth of antisepticism universally accepted, universally understood, and universally practised throughout British medicine? It is for our annual parliament, the meeting of the British Medical Association, to give us a true answer to this question. If it be proved on inquiry that this great practical advance in medical science is still in the stage of mere theory to the majority of us, then it is the urgent duty of our Association forthwith to organise a practical propaganda for the dissemination of elementary medical and surgical science among those who are charitably supposed to be past masters in the medical and surgical arts.

